# Cytokine Release Syndrome Is an Independent Risk Factor Associated With Platelet Transfusion Refractoriness After CAR-T Therapy for Relapsed/Refractory Acute Lymphoblastic Leukemia

**DOI:** 10.3389/fphar.2021.702152

**Published:** 2021-07-23

**Authors:** Yadan Liu, Bin Liang, Yan Liu, Guoqing Wei, Wenjun Wu, Luxin Yang, Li Yang, He Huang, Jue Xie, Yongxian Hu

**Affiliations:** ^1^ Bone Marrow Transplantation Center, The First Affiliated Hospital, School of Medicine, Zhejiang University, Hangzhou, China; ^2^ Zhejiang Province Engineering Laboratory for Stem Cell and Immunity Therapy, Hangzhou, China; ^3^ Institute of Hematology, Zhejiang University, Hangzhou, China; ^4^ Department of Hematology, Wenzhou Medical University, Wenzhou, China; ^5^ Department of Blood Transfusion, The First Affiliated Hospital, School of Medicine, Zhejiang University, Hangzhou, China

**Keywords:** chimeric antigen receptor T cells, platelet transfusion refractoriness, cytokine release syndrome, relapsed/refractory, acute lymphoblastic leukemia

## Abstract

**Background:** Chimeric antigen receptor T cell (CAR-T) therapy is successful in improving treatment outcomes for relapsed/refractory acute lymphoblastic leukemia (R/R ALL). However, toxicities associated with CAR-T therapy are being increasingly identified. Pancytopenia is one of the most common complications after CAR-T therapy, and platelet transfusions are an essential part of its supportive care.

**Study Design and Methods:** This study aimed to assess the effectiveness of platelet transfusions for R/R ALL patients at our single center and identify associated risk factors. Overall, 44 R/R ALL patients were enrolled in this study, of whom 26 received CAR-T therapy and 18 received salvage chemotherapy.

**Result:** Patients in the CAR-T group had a higher incidence of platelet transfusion refractoriness (PTR) (15/26, 57.7%) than those in the chemotherapy group (3/18, 16.7%) (*p* = 0.007). For patients receiving CAR-T therapy, multivariate analysis showed that the grade of cytokine release syndrome (CRS) was the only independent risk factor associated with PTR (*p* = 0.007). Moreover, higher peak serum IL-6 and IFN-γ levels suggested a higher risk of PTR (*p* = 0.024 and 0.009, respectively). Patients with PTR received more platelet infusion doses than those without PTR (*p* = 0.0426). Patients with PTR had more grade 3–4 bleeding events than those without PTR (21.4 vs. 0%, *p* = 0.230), and the cumulative incidence of grade 3–4 bleeding event was different (*p* = 0.023).

**Conclusion:** We found for the first time that PTR is associated with the CRS grade. Improved knowledge on the mechanisms of PTR after CAR-T therapy is needed to design a rational therapeutic strategy that aims to improve the efficiency of transfusions.

## Introduction

Patients with relapsed/refractory acute lymphoblastic leukemia (R/R ALL) usually have a very poor prognosis after salvage chemotherapy, with a median overall survival (OS) of 3–9 months (Zhang et al., 2018). Chimeric antigen receptor T-cell (CAR-T) therapy has revolutionized treatment modalities for R/R ALL with a complete remission (CR) rate of 70–90% (Maude et al., 2014; DasGupta et al., 2018; Wei et al., 2018). In our previous clinical trial of CD19-targeted CAR-T therapy for R/R ALL, a CR rate of 92.3% was achieved (Wei et al., 2018). We believe that increasing number of patients will benefit from CAR-T therapy in the coming years.

Despite the incredible therapeutic efficacy of CAR-T therapy, toxicities unique to CAR-T therapy, including cytokine release syndrome (CRS), B-cell aplasia, CAR-T-cell-related encephalopathy syndrome, infection, and pancytopenia, are being increasingly identified (Gödel et al., 2018; Shimabukuro-Vornhagen et al., 2018; Brudno and Kochenderfer, 2019; Cordeiro et al., 2020). Pancytopenia is one of the most common complications following CAR-T therapy for R/R ALL. After CAR-T therapy, 53% of patients had a grade 3 or 4 platelet count decrease, which greatly increases the risk of hemorrhage especially when important organs are involved (Wang et al., 2020). Therefore, for prophylactic or therapeutic reasons, platelet transfusions are an essential part of the supportive care for patients undergoing CAR-T therapy.

Platelet transfusion refractoriness (PTR) represents a significant clinical problem that complicates the provision of platelet transfusions and may be associated with increased hemorrhagic complications (Kerkhoffs et al., 2008). The causes of PTR are mainly associated with immune-related causes (alloimmunization to human leukocyte antigen [HLA], lymphocytotoxic antibodies, etc.), and non-immune-related causes (infection, high fever, sepsis, graft-versus-host disease, etc.) (Doughty et al., 1994; Slichter et al., 2005; Hod and Schwartz, 2008; Slichter et al., 2011; Comont et al., 2017). Other studies have focused on the rates and risk factors of PTR in patients undergoing chemotherapies or hematopoietic stem cell transplantation (HSCT) and have revealed that PTR is closely associated with lower CD34 ^+^ cell dose-infused and anti-HLA I antibodies (Solves et al., 2018; Tanoue et al., 2018). In addition, patients with extramedullary disease, low white blood cell (WBC) counts, infection, or hemophagocytic syndrome had a higher risk of developing PTR (Comont et al., 2017). After CAR-T therapy, patients usually have high fever, high serum cytokine levels, and low WBC counts. However, whether these patients have PTR after their CAR-T therapy remains elusive.

Given the lack of evidence, this study aimed to assess the effectiveness of platelet transfusions for R/R ALL patients who received CAR-T therapy at our single center and to identify associated risk factors.

## Materials and methods

### Patients

This retrospective study was registered (Chinese Clinical Trial Registry number, ChiCTR-ORN-16008948) and approved by the ethics committee of the First Affiliated Hospital, School of Medicine, Zhejiang University. We retrospectively analyzed the data of 44 R/R ALL patients between July 2011 and June 2019 at our single center. The patients were divided into a chemotherapy group and a CAR-T group. Patients’ enrollment is shown in Table 1. Eighteen patients received only salvage chemotherapy and comprised the chemotherapy group, and 26 patients received CAR-T therapy and comprised the CAR-T group. All R/R ALL patients underwent multiple first- or second-line chemotherapies.

**TABLE 1:** Patient base-line characteristics.

Characteristics	Chemotherapy	CAR-T therapy	*p* value
N	18	26	—
Age (years)	35.5 (16–67)	26 (15–66)	0.181
Sex	—	—	0.911
Male	8 (44.4%)	12 (46.2%)	—
Female	10 (55.6%)	14 (53.8%)	—
BSA	1.66 ± 0.17	1.63 ± 0.17	0.634
Bone marrow blasts	—	—	1.000
<20%	2 (11.1%)	2 (7.7%)	—
≥20%	16 (88.9%)	24 (92.3%)	—
Mutation	—	—	0.342
Normal	11 (61.1%)	16 (61.5%)	—
BCR-ABL	6 (33.3%)	4 (15.4%)	—
MLL	1 (5.6%)	3 (11.5%)	—
Other abnormalities	0 (0%)	3 (11.5%)	—
Cycles of prior therapy	—	—	0.968
<2	3 (16.7%)	3 (11.5%)	—
≥2	15 (83.3%)	23 (88.5%)	—
Duration of first remission	—	—	0.350
<1 year	14 (77.8%)	24 (92.3%)	—
≥1 year	4 (22.2%)	2 (7.7%)	—
No.relapse	—	—	0.540
<2	8 (44.4%)	14 (53.8%)	—
≥2	10 (55.6%)	12 (46.2%)	—
Transplantation	—	—	1.000
No	14 (77.8%)	21 (80.8%)	—
Yes	4 (22.2%)	5 (19.2%)	—
Extramedullary involvement	—	—	0.911
No	10 (55.6%)	14 (53.8%)	—
Yes	8 (44.4%)	12 (46.2%)	—
Infection	—	—	0.359
No	8 (44.4%)	16 (61.5%)	—
Yes	10 (55.6%)	10 (38.5%)	—

### Enrollment Standard

1). acute B-lineage lymphocytic leukemia, 2). primary refractory after induction chemotherapy, or relapse after remission, 3). patients with records of platelet transfusion at least 2 times after salvage chemotherapy or CAR-T treatment.

### Treatment Protocols

Patients in the chemotherapy group received one of the following five regimens: VMCP; VICP; Hyper-CVAD regimen A or B; FLAG; or MTX + Ara-C. These regimens are commonly used for treating R/R ALL and were selected by investigators based on patients’ conditions and treatment histories. The investigators and patients’ medical needs determined which supportive measures they required for optimal medical care. Regarding the CAR-T group, the treatment protocol mainly comprised a conditioning regimen (consisting of 2 days of cyclophosphamide 0.5 g/m^2^ and 3 days of fludarabine 30 mg/m^2^/d) and CAR-T cell infusion, as described previously (Shank et al., 2017; Brudno and Kochenderfer, 2018). CAR-T cells were infused after 1 day interval of finishing conditioning regimen.

### Supportive Care

Patients received PLT transfusions if their PLT levels dropped below 20 × 10^9^/L. All transfused platelets were filtered and irradiated with gamma rays (at least 25 Gy), were stored for no more than 48 h, and were ABO-compatible with the transfused patients. Patients underwent blood routine examinations within 12 h after receiving PLT transfusions. Other supportive care, such as red blood cell transfusions if their hemoglobin levels were <60 g/L, G-CSF (5 μg/kg/d) was administered to all patients if the neutrophil levels dropped below 0.5 × 10^9^/L, and they received G-CSF until the levels were higher than 2.0 × 10^9^/L. In the duration of CAR-T cell therapy, tocilizumab (humanized monoclonal antibody against IL-6 receptor) or corticosteroid were administered for supportive treatment for some severe patients.

### Definitions

PTR is defined as a failure increment after platelet transfusion. It should be made only when at least two transfusions of ABO-compatible units, stored for <72 h, have a corrected count increment (CCI) of <5,000/μL simultaneously (Hod and Schwartz, 2008; Schiffer et al., 2018; Solves et al., 2018). We used the 12-h CCI to assess whether a patient had PTR (Du Bois and Du Bois, 1989; Davis et al., 1999; Jia et al., 2014; Solves et al., 2018). The CCI is calculated using the following formula:

CCI = (post-transfusion platelet count - pre-transfusion platelet count (/μL)) × body surface area (BSA) (m^2^) ÷ number of platelets transfused (10^11^).

BSA (Du Bois formula) = 0.00718 × height^0.725^ (/cm) × weight^0.425^ (/kg).

Bleeding severity was assessed according to the WHO bleeding scale after CAR-T cell infusion (Stanworth et al., 2013).

CRS is defined as a life-threatening systemic inflammatory response that can be triggered by CAR-T cell infusion and is associated with an elevated cytokine (IL-6 and IFN-γ) level. The CRS grade system after CAR-T therapy was assessed based on previous research (Lee et al., 2014; Gödel et al., 2018).

### Statistical Analysis

The Shapiro-Wilk test was used to assess if continuous data conformed to the normal distribution pattern. Continuous data are presented as mean ± SD and median range; categorical data are presented as numbers and percentages. The differences in continuous data and those in categorical data between the two groups were compared using Mann-Whitney U test and the Chi-square test or Fisher’s exact test, respectively. The multivariate analysis for factors associated with PTR was performed using binary logistic regression in the CAR-T group; it included some available variables (*p* <0.30 in univariate analysis), and chose forward LR method. The cumulative incidence of bleeding after CAR-T cell infusion was calculated using R version 3.6.1and R studio. All *p* values were two-sided, and the results with *p* values <0.05 were considered statistically significant. Computer software (SPSS, Version 26.0, SPSS Software, Inc. Chicago, IL) or GraphPad Prism (6.0r version 3.6.1) was used for all statistical analyses.

## Results

### Patient Characteristics

Overall, 44 R/R ALL patients aged 15–67 years were enrolled in this study. Twenty-six patients received CAR-T therapy and 18 received salvage chemotherapy. All data were collected before patients underwent their latest chemotherapy or CAR-T cell infusion. Baseline clinical characteristics of all patients are shown in Table 1. The median age of patients in the CAR-T group was 26 (15–66 years), 46.2% of patients were men, the median age of patients in the chemotherapy group was 35.5 (16–67 years), and 44.4% of patients were men. There were no statistically significant differences between the CAR-T and chemotherapy groups with respect to age, gender, BSA, blast cell proportions in bone marrow, number of previous induction chemotherapy episodes, mutation, duration of time since the first remission, and number of relapses, infection, patients with/without extramedullary involvement, and patients who did/did not undergo transplantation (*p* > 0.05).

### Incidence of Platelet Transfusion Refractoriness in Patients in the Chimeric antigen receptor T cell and Chemotherapy Groups

Eighteen patients had PTR. More importantly, three patients (16.7%) in the chemotherapy group and 15 patients (57.7%) in the CAR-T group had PTR. There was a statistically significant difference between the two groups (χ^2^ = 7.406, *p* = 0.007) (Table 2), suggesting a higher risk of PTR in the CAR-T group.

**TABLE 2:** Comparison of PTR incidence between chemotherapy group and CAR-T group.

	Chemotherapy	CAR-T therapy	χ^2^	*p* value
Non-PTR	15 (83.3%)	11 (42.3%)	7.406	0.007
PTR	3 (16.7%)	15 (57.7%)		

### Complete Remission Rate Between Chimeric antigen receptor T cell and Chemotherapy Groups

Among 26 patients in the CAR-T group, one patient with PTR lacked a detailed cytokine profile, we excluded it and 25 patients were evaluated. Patients in CAR-T group were divided into Non-PTR group and PTR group according to whether two consecutive CCI <5,000/μL. The data of 25 patients were analyzed in Table 3. After CAR-T treatment, 72% (18/25) of the patients achieved CR, of which 9 (64.3%) patients in the PTR group, and 9 patients (81.8%) in the Non-PTR group, the *p* value determined by Fisher’s exact test was 0.407, there was no significant difference in CR rate between the two groups.

**TABLE 3:** Patient characteristics after CAR-T treatment.

Characteristics	Non-PTR	PTR	*p* value
N	11	14	
Age (years)	25 (16–66)	29 (15–65)	0.979
Sex	—	—	0.695
Male	6 (54.5%)	6 (42.9%)	—
Female	5 (45.5%)	8 (57.1%)	—
BSA (kg/m^2^)	1.65 (1.35–1.86)	1.57 (1.42–1.93)	0.851
WBC count (10^9^) before CAR-T	6.4 (1.8–38.3)	3.95 (0.2–75.1)	0.373
Hb count (g/L) before CAR-T	77.27 ± 34.83	84.36 ± 22.07	0.541
Platelet count (10^9^) before CAR-T	75.73 ± 54.602	71.50 ± 63.92	0.863
Bone marrow blasts before CAR-T	—	—	0.689
<50%	4 (36.4%)	7 (50.0%)	—
≥50%	7 (63.6%)	7 (50.0%)	—
Bone marrow blasts percentage (median)	70% (6–84%)	58% (0–92%)	0.647
Gene mutation	—	—	0.645
Normal	6 (54.6%)	9 (64.3%)	—
BCR-ABL	1 (9%)	3 (21.4%)	—
MLL arrangement	2 (18.2%)	1 (7.1%)	—
Other abnormalities	2 (18.2%)	1 (7.2%)	—
Cycles of prior therapy	—	—	1.000
<3	5 (45.5%)	7 (50.0%)	—
≥3	6 (54.5%)	7 (50.0%)	—
Duration of first remission	—	—	0.487
<1 year	11 (100%)	12 (85.7%)	—
≥1 year	0 (0%)	2 (14.3%)	—
No. of relapse	—	—	1.000
<2	6 (54.5%)	7 (50.0%)	—
≥2	5 (45.5%)	7 (50.0%)	—
Prior allo-HSCT	—	—	0.288
No	8 (72.7%)	13 (92.9%)	—
Yes	3 (27.3%)	1 (7.1%)	—
Extramedullary involvement	—	—	0.695
No	5 (45.5%)	8 (57.1%)	—
Yes	6 (54.5%)	6 (42.9%)	—
Complete remission	—	—	0.407
No	2 (18.2%)	5 (35.7%)	—
Yes	9 (81.8%)	9 (64.3%)	—
No.of tocilizumab	—	—	0.241
0	6 (54.5%)	4 (28.6%)	—
1	1 (9.1%)	4 (28.6%)	—
2	4 (36.4%)	6 (42.9%)	—
Corticosteroid	—		0.208
No	9 (81.8%)	7 (50%)	—
Yes	2 (18.2%)	7 (50%)	—
Improvement of neutrophil deficiency within 30 days	—	—	1.000
No	5 (45.5%)	6 (42.9%)	—
Yes	6 (54.4%)	8 (57.1%)	—
Recovery of neutrophil deficiency within 3 months	—	—	0.180
No	1 (9.1%)	5 (35.7%)	—
Yes	10 (90.9%)	9 (64.3%)	—
Improvement of platelet within 30 days	—	—	1.000
No	8 (72.7%)	10 (71.4%)	—
Yes	3 (27.3%)	4 (28.6%)	—
Recovery of platelet within 3 months	—	—	0.033
No	1 (9.1%)	8 (57.1%)	
Yes	10 (90.9%)	6 (42.9%)	
Infection			0.099
No	9 (81.8%)	6 (42.9%)	—
Yes	2 (18.2%)	8 (57.1%)	—
Highest temperature during CAR-T treatment (°C)	39.96 ± 0.69	40.54 ± 0.63	0.039
Highest level of C-reaction protein during CAR-T treatment (mg/L)	142.41 ± 104.10	149.47 ± 58.67	0.843
CRS grade	—	—	0.005
≤2	8 (72.7%)	2 (14.3%)	—
≥3	0 (0%)	3 (21.4%)	—

### Use of Antibody of IL-6 Receptor

Some patients with severe CRS were treated with tocilizumab and corticosteroid after CAR-T cell infusion. We found that 10 (40%) of 25 patients did not use tocilizumab, 5 patients (20%) used 1 time, 10 patients (40%) used 2 times. of which 6 cases (42.9%) used 2 times in the PTR group, compared with 4 cases (36.4%) in Non-PTR group. Between PTR group and Non-PTR group, whether or not to use tocilizumab, the *p* value was 0.241, there was no significant statistical difference.

### Use of Corticosteroid

Among 25 patients, 16 cases (64%) were not used corticosteroid, 9 cases (36%) were used, of which 7 cases (50%) were used in the PTR group, only 2 cases (18.2%) in the Non-PTR group. The *p* value calculated by the chi-square test was 0.208. There was no significant statistical difference between two groups.

### Improvement and Recovery of Neutrophil Deficiency

Most patients undergoing CAR-T cell therapy in our center have been hospitalized for approximately one month, so the blood routine observed time is set 30 days. When patients have agranulocytosis, they will be treated with granulocyte colony stimulating factor for long-term treatment immediately.

The improvement of neutrophils is > 0.5 × 10^9^/L for more than 3 consecutive days since agranulocytosis. Neutrophils recovery is that the count rises to normal levels.

Among the 25 patients, a total of 14 patients changed from agranulocytosis to non-agranulocytosis within 30 days 8 cases (57.1%) achieved neutrophils improvement in the PTR group, and 6 cases achieved neutrophils recovery (54.5%) in the Non-PTR group, In terms of the improvement of neutrophil deficiency within 30 days after CAR-T cell treatment, the *p* value was 1.000, implied that there was no statistical difference between the two groups.

After 3 months of blood routine follow-up, we found that 19 of 25 patients eventually achieved neutrophil recovery. 9 patients (64.3%) in the PTR group, and 10 patients (90.9%) in the Non-PTR group. the *p* value was 0.180. Because some patients died or were lost to follow-up, the blood routine could not be traced back to a longer time.

### Improvement and Recovery of Thrombocytopenia

The improvement of platelet is that the patient's platelet stabilized above 20 × 10^9^/L for 7 consecutive days without platelet transfusion since below 20 × 10^9^/L. The recovery of platelets is the platelet count back to normal levels.

Within 30 days, 7 of 25 patients achieved thrombocytopenia improvement, of which 4 patients (28.6%) in the PTR group, and 3 patients (27.3%) recovered in the Non-PTR group. In terms of improvement of thrombocytopenia within 30 days after CAR-T cell treatment, *p* value of 1.000, there was no significant difference between two groups.

After 3 months of blood routine follow-up, 6 cases (42.9%) in the PTR group recovered to normal platelet, 10 cases (90.9%) recovered in the Non-PTR group, The *p* value was 0.033, indicating that PTR has a continuous negative effect on the subsequent platelet recovery.

### Independent Risk Factors Associated With Platelet Transfusion Refractoriness

The CAR-T group patients were divided into a Non-PTR group and a PTR group based on whether two continuous CCI measurements were <5,000 simultaneously. Deep analysis of the data of the 25 patients was performed (Table 3). Among 10 (40%) patients with CRS grade ≤2, only two (20%) developed PTR, whereas among 15 (60%) patients with CRS grade ≥3, 12 (80%) developed PTR. Because of an insufficient sample size, Fisher’s exact test was performed. *p* value of 0.005 indicated that patients with CAR-T cell infusion had a higher risk of PTR among patients with a high CRS grade.

In addition, we observed that the peak body temperature of PTR group patients was higher than that of Non-PTR group patients (t = -2.189 and *p* = 0.039), which indicated a statistically significant difference between the two groups. The average peak body temperature in the PTR group was higher than that in the Non-PTR group. During the CAR-T cell treatment, 10 patients (40%) also developed infection, of which 8 patients (57.1%) were infected in the PTR group, and 2 patients (18.2%) in the Non-PTR group, *p* value of 0.099 is no indicated that there was not statistical difference.

**FIGURE 1:**
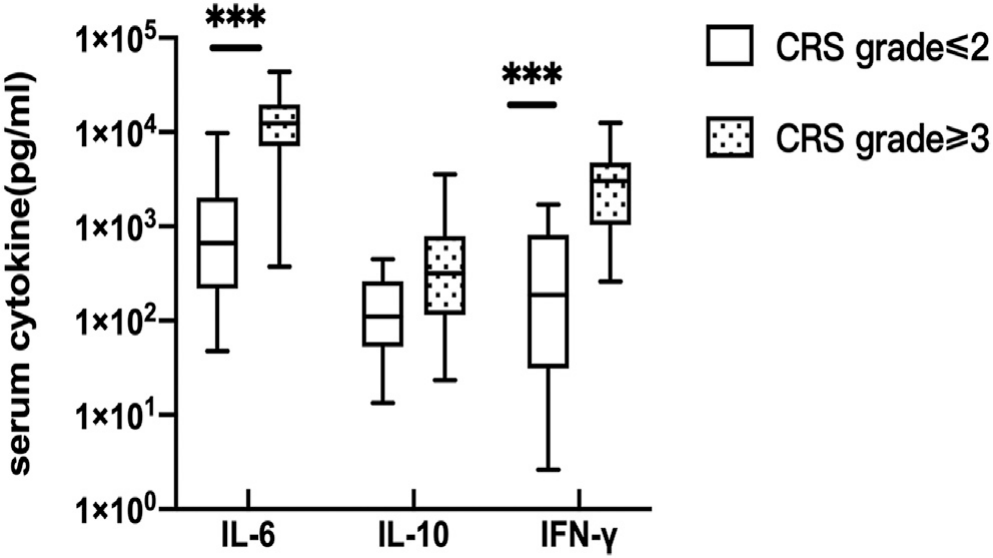
Correlation of serum cytokine levels and CRS. the peak serum cytokine, respectively IL-6 (*p* = 0.001) and IFN-γ(*p* = 0.001) were significantly different between CRS grade≤ 2 and CRS grade≥ 3.

Serum peak cytokine levels were classified according to the CRS grade. The median (range) IL-6, IL-10, and IFN-γ in CRS grade≤ 2 and CRS grade≥ 3 were 662.055 (47.570–9726.050) vs. 12,458.750 (374.900–43,753.920) pg/ml, 110.540 (13.350–448.310) vs. 317.41 (23.480–3556.740) pg/ml, and 186.770 (2.630–1701.310) vs. 3,018.200 (258.570–12,557.570) pg/ml, respectively. The *p* values of serum IL-6, IL-10, and IFN-γ were 0.001, 0.090, and 0.001, respectively (Figure 1). The difference was statistically significant, indicating that patients with higher CRS grade have higher serum cytokine levels (IL-6 and IFN-γ).

We performed a multivariate analysis of other potential factors (*p* ≤ 0.05) affecting PTR using binary logistic regression (Table 4), which included some available clinical factors (prior allo-HSCT, infection) in the univariate analysis. Based on the results, we could infer that the CRS (OR = 16, 95%CI 2.165–118.270, *p* = 0.007) was an independent factor contributing to PTR in patients receiving CAR-T therapy.

**TABLE 4:** Multivariate analysis of factors influencing PTR.

Factors	OR	95%CI	*p* value
CRS grade	16	2.165–118.270	0.007
IL-6			0.642
IFN-γ			0.281
Infection			0.194
Prior allo-HSCT			0.171
Highest temperature during CAR-T treatment (°C)			0.158

### Correlation Between Serum IL-6 and IFN-γ Levels and Platelet Transfusion Refractoriness

**FIGURE 2:**
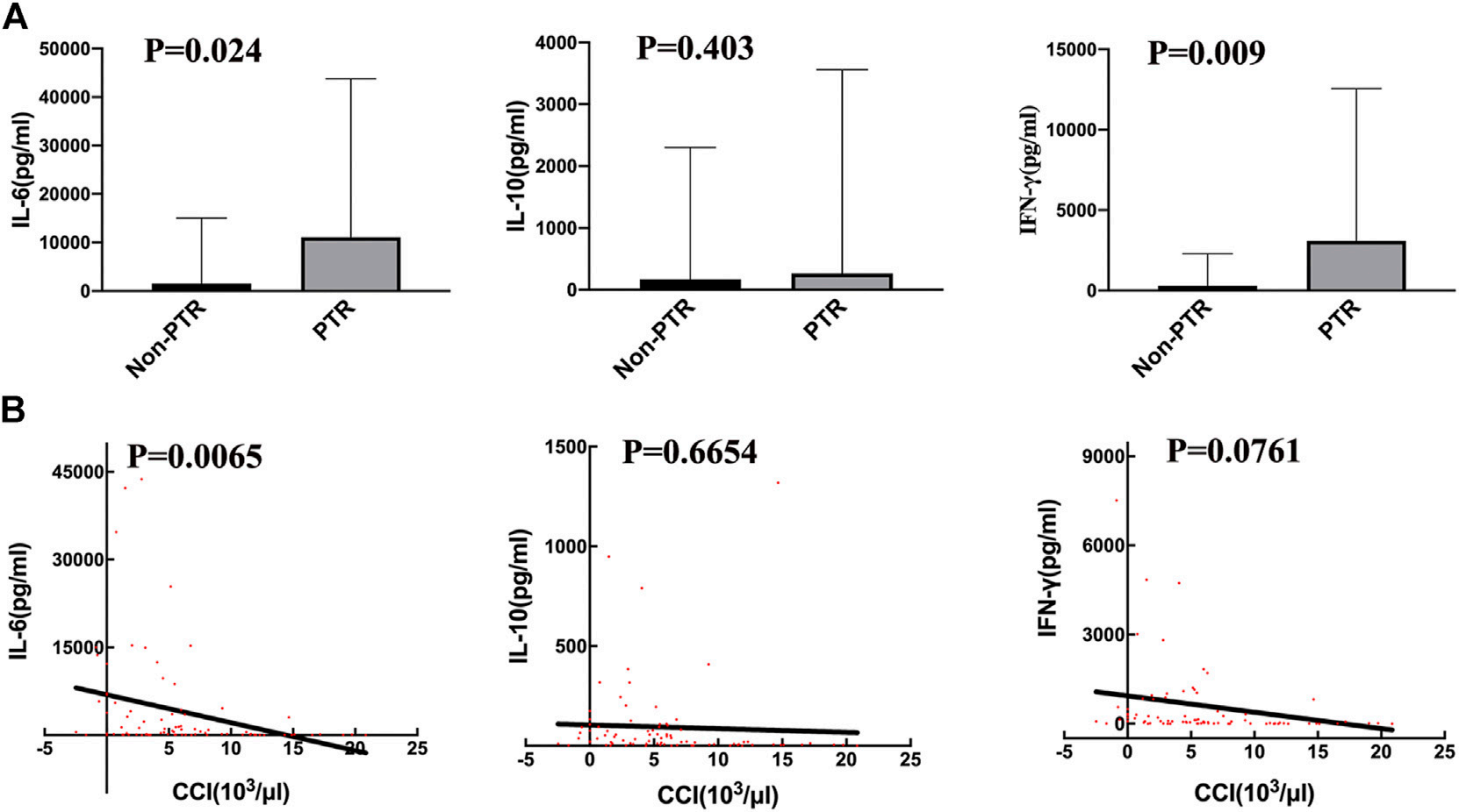
Correlation of serum cytokine levels and PTR. **(A)** the peak serum cytokine, respectively IL-6 (*p* = 0.024) and IFN-γ (*p* = 0.009), levels are significantly different between the PTR and Non-PTR groups. **(B)** The values of 12 h CCI with corresponding serum cytokine, were negatively related to serum IL-6 level (*p* = 0.0065).

Serum cytokine levels are usually increased during CRS. The peak cytokine levels in the PTR group were higher than those in the Non-PTR group (Figure 2A), and the median (range) IL-6, IL-10, and IFN-γ levels for the two groups were 11,101.39 (374.9–43,754) vs. 1,531.15 (47.57–15,071) pg/ml, 266.25 (23.48–3557) vs. 171.01 (13.35–2302) pg/ml, and 3,093.82 (113.80–12,558) vs. 296.49 (2.630–2288) pg/ml, respectively. The *p* values for serum IL-6 and IFN-γ were 0.024 and 0.009 respectively, showing a statistical significance between the two groups. According to the results, higher serum IL-6 and IFN-γ levels suggested a higher risk of developing PTR.

We used CCI to evaluate transfusion efficiency after every transfusion. A linear regression analysis was performed on CCI and corresponding cytokine levels on the day of transfusion (Figure 2B). The results revealed that only IL-6 was negatively associated with CCI (*p* = 0.0065, R square = 0.09). Thus, serum cytokines IL-6 and IFN-γ were related to PTR; however, IL-6 may have a stronger effect on developing PTR than IFN-γ.

### Association Between Platelet Transfusion Refractoriness and Doses of Platelet Transfusion

**FIGURE 3:**
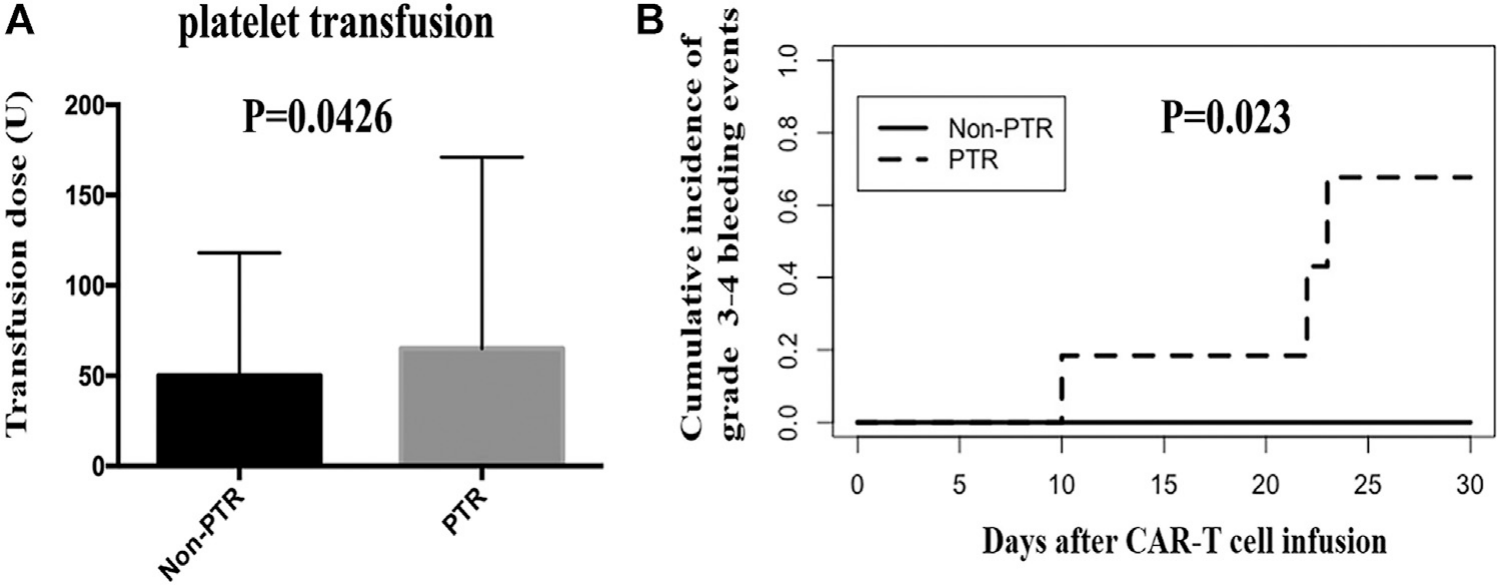
Platelet transfusion and grade 3–4 bleeding events after CAR-T therapy. **(A)** the units of platelet transfusion was significantly different between PTR and Non-PTR group (*p* = 0.0426). **(B)** Cumulative incidence of grade 3–4 bleeding events in PTR and Non-PTR group was significantly different between PTR and Non-PTR group (*p* = 0.023).

We calculated the total platelet transfusion units for 25 patients who received CAR-T cell infusion until their platelet counts were up to 20 × 10^9^/L. The median units of platelet transfusion in the Non-PTR and PTR groups were 50 (25–86) and 65.5 (40–171) units, respectively. A statistically significant difference was found between the two groups (*p* = 0.0426) (Figure 3A). The results implied that PTR patients received more units of platelet infusion.

### Grade 3–4 Bleeding Events in Patients in the Platelet Transfusion Refractoriness and Non-Platelet Transfusion Refractoriness Groups

In the CAR-T group, we observed 25 patients within 30 days of receiving CAR-T cell infusion to identify whether patients experienced a bleeding event. Six patients (24%) had a grade 0 bleeding event. Ten (40%), four (16%), two (8%), and three (12%) patients had a grade 1 bleeding event, grade 2 bleeding event, grade 3 bleeding event, and grade 4 bleeding event, respectively. Importantly, three patients with a grade 4 event were from the PTR group and died of intracranial hemorrhage, pulmonary hemorrhage, and gastrointestinal hemorrhage. The cumulative incidence of grade 3–4 bleeding event was different in the PTR and Non-PTR groups, 67% vs. 0% at 30 days, (*p* = 0.023). Patients in the PTR group were more likely to have grade 3–4 bleeding events than those in the Non-PTR group after CAR-T therapy (Figure 3B).

## Discussion

This study retrospectively observed 44 R/R ALL patients undergoing salvage chemotherapies or CAR-T therapy and aimed to clarify the incidence and risk factors associated with PTR in patients after CAR-T therapy. Based on our study, we found the incidence of PTR in the CAR-T group was significantly higher than that in the chemotherapy group, which has not been previously reported. Moreover, CRS was an independent risk factor associated with PTR, whereas serum IL-6 and IFN-γ levels during CRS were positively associated with PTR.

The incidence of PTR in patients with acute myeloid leukemia receiving induction chemotherapies was slightly different in our study compared with that in previous research (Trial to Reduce Alloimmun, 1997; Comont et al., 2017). In the Trial to Reduce Alloimmunization to Platelets study group, 51 (10%) of 530 patients became refractory to platelet transfusions. In the largest study of first-line intensive chemotherapy or patients with AML, 41 (4.8%) of 897 patients had PTR. The proportion of PTR in children with ALL was only 2.3% (DeCoteau et al., 1995). The incidence of PTR in patients with acute leukemia undergoing chemotherapy was <20%. Patients with R/R ALL usually have a high risk of PTR because of multiple blood transfusions. However, the incidence of PTR was significantly higher in patients with R/R ALL (57.7%) undergoing CAR-T therapy than in those undergoing salvage chemotherapy (16.7%) in our study, implying the existence of different mechanisms. Therefore, it is especially important to clarify risk factors and underlying mechanisms associated with PTR after CAR-T therapy.

To determine the causes of the high incidence of PTR in the CAR-T patient group, we analyzed the characteristics of the 25 R/R ALL patients who received CAR-T therapy. More patients with CRS grades 3–4 developed PTR than those with CRS grades 1–2. The pathogenesis of PTR after CAR-T therapy is unknown and may be multifactorial. CRS is the most common toxicity after CAR-T therapy. It is triggered by the activation of CAR-T cells with cognate antigens expressed by tumor cells. The activated CAR-T cells release cytokines (IL-6, IL-10, and IFN-γ), as do other bystander immune cells, such as monocytes, and/or macrophages, and dendritic cells.

Based on some researches about CRS, it was found that IL-6 is actually the central mediator of CRS toxicity. The presence of high levels of IL-6 in the CRS may initiate the pro-inflammatory signal cascade mediated by IL-6 (Lee et al., 2014). Although studies have shown that there is a close relationship between inflammatory factors and the severity of CRS, the accuracy of predicting the severity of CRS in patients based on cytokine levels is still unclear (Davila et al., 2014). Our research shows that PTR is positively correlated with CRS grade. In addition, the peak cytokine (IL-6 and IFN-γ) levels in the PTR group were higher than those in Non-PTR group, and CCI was negatively correlated with the serum cytokine IL-6 levels, implying that IL-6 contributed to CRS and was the more important factor resulting in PTR. At the same time, patients with higher CRS grade also have higher serum cytokine levels, which shows that there is a close correlation among PTR, CRS grade, and cytokine levels. However, the current classification of CRS grade is mainly based on clinical symptoms and is not included serum cytokine level, further studies are needed to clarify the detailed mechanisms.

Some studies have proved that infection is actually also a factor that easily triggers PTR (Doughty et al., 1994; Slichter et al., 2005), but our study did not found the obvious correlation between infection and PTR. Due to the small sample in this trial, perhaps CRS is a more important factor that causes PTR after CAR-T treatment, and then reducing the role of infection in the pathogenesis of PTR.

According to research on CAR-T therapy, in coagulation disorders (even disseminated intravascular coagulation), platelet reduction occurs during CRS (Mei et al., 2018; Jiang et al., 2019; Wang et al., 2020). Given this, patients receiving CAR-T therapy are at a high risk of having vital organ hemorrhages as a complication, which can be life-threatening. Our study also observed this association with platelet levels decreasing and organs hemorrhaging. Most patients who received CAR-T cell infusions had varying degrees of bleeding. Interestingly, compared with patients without PTR, only those with PTR had an extreme severe bleeding event, leading to mortality.

Of the total 1,846 platelet transfusion units administered to 25 adults, the PTR group had 1,249 units and the Non-PTR group had only 597 units, with a statistical difference between the two groups. To date, effective preventive and therapeutic strategies to combat the complications of PTR after CAR-T therapy remain unknown. During the CAR-T cell treatment in our study, two patients with PTR both received one cross-match-compatible platelets transfusion, then successfully achieved temporary satisfactory response on platelet transfusion compared with before. CAR-T cell therapy is a treatment that reduces antibodies by destroying B cells, we predict that Anti-HLA class I antibodies is not the main factor for the ineffective platelet transfusion after CAR-T therapy. Besides, 4 patients used TPIAO, 3 patients used Recombinant Human Interleukin-11 for Injection, 1 patient received Intravenous immunoglobulin (IVIG) injection, these strategies were no significant improvement in platelets count and the effectiveness of platelet transfusion, other studies also implied that current therapeutic strategies were not always effective, such as thrombopoietin receptor agonists (TPO-RA), splenectomy, plasma exchange, rituximab, and IVIG (Yu et al., 2015; Platelet Transfusion for Patients With Cancer, 2018; Berthelot-Richer et al., 2012; Cid et al., 2015; Mauro et al., 2016). Because of close correlation between serum cytokine levels and PTR, Timely use of tocilizumab to control serum cytokine levels or plasma exchange may be effective in controlling the CRS and improving the ineffectiveness of platelet transfusions. By tracking the recovery of neutrophils and platelets, we found that patients in PTR group can have a more long-term negative impact on the recovery of platelets. although there are different views on whether fever increases the risk of bleeding in humans (de la Serna et al., 2008; Gerber et al., 2008; Stanworth et al., 2015), it is still recommended platelet transfusion when patients have infection or fever with platelet count below 20 × 10^9^/L. Therefore, in the absence of a recognized mechanism-based treatment strategy, we suggest platelet transfusion as an important supportive treatment for CAR-T cell therapy.

This study had some limitations, including its retrospective nature and the limited sample size, which might affect the reliability of the statistical analysis. The choice of covariates for the multivariate analysis was constrained by the small number of observed events. The mechanisms underlying CRS and PTR were also not clearly demonstrated.

In conclusion, for the first time, we found that PTR was associated with the CRS grade. Furthermore, PTR was a severe condition associated with a high risk of death from bleeding. Therefore, improved knowledge on the mechanisms of PTR after CAR-T therapy is needed to design a rational therapeutic strategy that aims to improve the efficiency and safety of transfusions. Overcoming PTR would improve conditions and prognosis of patients receiving CAR-T therapy.

## Data Availability

The raw data supporting the conclusion of this article will be made available by the authors, without undue reservation.
